# Development of a 3D tumor model based on decellularized matrix using high-throughput approaches

**DOI:** 10.3389/fbioe.2025.1690844

**Published:** 2026-01-20

**Authors:** Homayoon Siahmansouri, Daniela Fenoglio, Gilberto Filaci, Maddalena Mastrogiacomo

**Affiliations:** 1 Department of Experimental Medicine (DIMES), University of Genova, Genova, Italy; 2 Department of Internal Medicine and Medical Specialities (DIMI), University of Genova, Genova, Italy; 3 IRCCS Ospedale Policlinico San Martino, Genoa, Italy

**Keywords:** cancer 3D model, decellularization, extracellular matrix, recellularization, tumor microenvironment

## Abstract

Precision medicine aims to develop 3D tumor models to validate new therapies and investigate disease mechanisms by isolating the extracellular matrix as a foundation for recreating tumors *in vitro*. The use of decellularized tumor matrices offers a promising and versatile platform for *in vitro* cancer research and therapeutic testing. The tumor microenvironment (TME) is the surrounding milieu of cancerous tissues and contains an intricate network of extracellular matrix (ECM) components and signaling proteins that regulate tumorigenesis, invasion, and metastasis. However, the decellularization techniques process can be disruptive and often damage essential macromolecules and proteins, potentially compromising the restoration of a biologically relevant microenvironment during recellularization. This review explores the most relevant macromolecules and proteins within the TME, emphasizing their roles in tumors and metastasis. Here, the potential of reinstating these components into decellularized tumor scaffolds to enhance their biological relevance and functionality is highlighted. Key macromolecules, including collagen, fibronectin, hyaluronic acid (HA), and laminin, are discussed alongside the contributions of proteins such as integrins, matrix metalloproteinases (MMPs), and growth factors to ECM remodeling, cell adhesion, migration, and proliferation. The strategic reintroduction of these elements will improve the recellularization process and create more realistic TME models. These improved models hold promise for cancer research, medication discovery, and therapeutic testing, providing a deeper understanding of tumor biology and enabling the development of more effective treatment strategies.

## 1 Introduction

The TME is responsible for tumor initiation, metastasis, and therapy resistance in various types of cancers (Quail and Joyce, 2013; Wang Y. et al., 2024). The TME is composed of a complex, three-dimensional ECM that offers structural support to tumor cells, allows for cell-to-cell communication, and contributes to regulating cellular functions such as adhesion, migration, and differentiation. Nevertheless, this dynamic microenvironment is disrupted during cancer progression, which induces modifications of the ECM, both its composition and mechanical properties (Winkler et al., 2020; Henke et al., 2020). The sponge-like decellularization of tumor tissues, which maintains the extracellular matrix (ECM) architecture and composition, is a novel and exciting method for studying cancer *in vitro*. To create a physiologically relevant tumor microenvironment (TME), this decellularized scaffold can subsequently be recellularized with tumor and stromal cells. Recellularization is aimed at creating a more accurate *in vitro* model that preserves patient-specific extracellular matrix components, like structural proteins and bioactive molecules, so that cancer cell behavior, drug response, and immune interactions can be studied in an environment that more closely resembles *in vivo* conditions (Garc et al., 2022; Li et al., 2025).

However, the challenge of decellularization lies in the ability to retain the macromolecules and ECM proteins that are required for preserving the native architecture of the tumor, as decellularization may unintentionally denature these proteins (Bhattacharya et al., 2024; Iazzolino et al., 2022). Major ECM components, including collagen, fibronectin, HA, and proteoglycans, are important in cell signaling and interactions and facilitate the adhesion, migration, and invasion of tumor cells (Heiss et al., 2025; Yang et al., 2025). Because these proteins can play important roles in mediating the re-establishment of native cell‒cell and cell‒matrix interactions within a scaffold, the loss of proteins during decellularization can have a drastic impact on recellularization. This is a considerable hurdle in the development of a viable 3D model of the TME for studying tumors and screening drugs (Fernández-Pérez and Ahearne, 2019).

Recent approaches have been aimed at enhancing the recellularization of such decellularized tissues by reloading them with tissue-relevant macromolecules and proteins desirable for cellular function (Fok et al., 2023; Das et al., 2020). In addition, these proteins can promote the upregulation of signaling pathways involved in tumor cell behavior and more accurately mimic the microenvironment with respect to the natural tumor stroma (Levental et al., 2009).

In this review, we describe the roles of these macromolecules and proteins in the context of cancer, which are important for the recellularization of decellularized tumor tissues. We summarize their roles in ECM remodeling and potential applications for enhancing 3D tumor models. We will further discuss the obstacles encountered in the decellularization procedure and the methods used to address these challenges to establish better quality and more physiologically relevant tumor structures suitable for both cancer studies and drug screening.

## 2 Decellularization and challenges

Decellularization is a complex procedure that removes living cells and cellular components from biological tissues, yielding an intact ECM. The ECM, which is composed of proteins, glycoproteins, and bioactive molecules, maintains the structural and functional properties of its corresponding native tissue and functions as an optimal 3D scaffold for cell recellularization (Crabbé et al., 2015; Liu et al., 2024). Decellularization has recently become one of the key technologies in the fields of tissue engineering and regenerative medicine, with promise for advancing both basic science research and translation to the clinic. Decellularization usually consists of a combination of physical, chemical, and enzymatic methods. Physical techniques such as freeze‒thaw cycles or agitation to dissociate cells can yield the removal of cellular material while preserving the ECM structure. Detergents and acidic or alkaline solutions solubilize cellular constituents, allowing their removal *via* chemical methods. Nucleases used as enzymatic treatments further degrade residual nucleic acids and cellular debris. The methods differ on the basis of the tissue type used, the end purpose of the ECM created, and the sensitivity of the ECM to this method (Keane et al., 2015). The main categories of decellularization methods, along with their relative advantages, limitations, and effects on ECM preservation, are summarized in Table 1.

**TABLE 1:** Decellularization methods.

Method	Details	Advantages	Disadvantages	Decellularization efficiency	ECM preservation	Cytotoxicity	Duration	References
Chemical methods	Use of detergents (e.g., SDS, Triton X-100), acids, or organic solvents to solubilize cells	Effective in removing cellular debris; suitable for dense tissues	Can damage ECM integrity; residues may cause cytotoxicity	High	Low–Moderate	High	Hours to days	White et al. (2017), Miranda et al. (2020)
Physical methods	Include freeze‒thaw cycles, hydrostatic pressure, and sonication	Simple and cost-effective; maintains ECM structure if optimized	Limited effectiveness for thick tissues; potential mechanical damage	Low–Moderate	High	Low	Minutes to hours	Wang et al. (2023), Azhim et al. (2014)
Enzymatic methods	Application of enzymes like trypsin, DNase, or RNase to degrade cellular components	Gentle on ECM; effective in nucleic acid removal	Time-consuming; may leave residual enzymes	Moderate	Moderate- High	Moderate	Hours to days	Gilpin and Yang (2017)
Combined techniques	Integration of chemical, physical, and enzymatic methods for enhanced efficiency	Maximizes cell removal and ECM preservation	Requires optimization; higher complexity and cost	High	Moderate	Moderate	Varies	Miranda et al. (2021)
Novel methods	Advanced techniques such as supercritical CO_2_ extraction and perfusion-based systems	Highly efficient; preserves ECM ultrastructure; eco-friendly	Expensive setup; requires specialized equipment	High	High	Low	Hours to days	Choi et al. (2024)

Decellularization is an essential step that allows for the preservation of native tissue architecture but removes cellular components that are largely immunogenic in nature. Decellularization can greatly decrease the risk of immune rejection by removing antigens and allows for the use of xenogeneic or allogeneic tissues in transplantation (Ott et al., 2008). Moreover, decellularized scaffolds are widely used in regenerative medicine for the repair and reconstruction of damaged tissues, including the skin, heart, liver, and kidneys. It serves as a natural scaffold directing cell repopulation and tissue regeneration and thus represents an effective alternative to conventional grafts. Decellularized scaffolds also play important roles in the study of development and cell biology processes such as cell differentiation, tissue morphogenesis, and organogenesis under relevant biological conditions (Neishabouri et al., 2022; Dussoyer et al., 2020).

**FIGURE 1:**
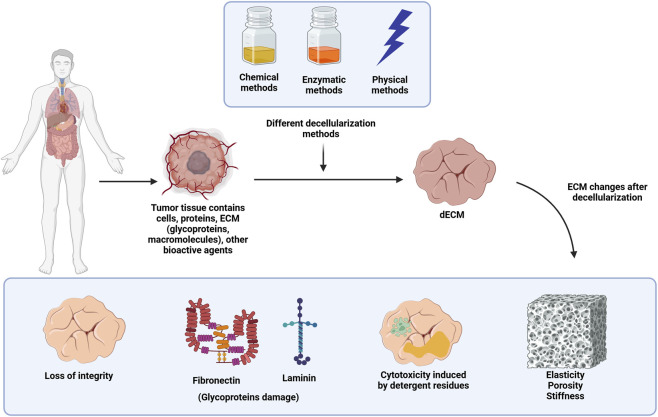
The figure indicates decellularization of tumor tissue using physical, chemical and enzymatic processes for the generation of decellularized ECM (dECM). Even though the immunogenic components like DNA, RNA, and cells are removed during decellularization, several changes could be triggered in the ECM, which include a lack of integrity, damage to major glycoproteins like fibronectin or laminin, detergent cytotoxicity, or differences in the elasticity, stiffness, or porosity of the ECM.

However, decellularization has many crucial challenges that must be solved to enhance its performance in tissue engineering and regenerative medicine. The challenge comes from the complicated nature of maintaining a functional structure close to the ECM while ensuring the complete removal of the cellular material. These include technical, biological and application-specific elements that largely affect the efficacy and reproducibility of decellularized scaffolds (Hussein et al., 2023). One of the main hurdles remains to ensure adequate cellular removal while maintaining ECM integrity. All cell debris, including DNA, RNA and membrane-associated proteins, needs to be fully removed to avoid immune rejection by the host (Uygun et al., 2010). Unfortunately, the protocols for decellularization, chemical, enzymatic, or physical, often result in the loss of ECM integrity. For example, detergents such as sodium dodecyl sulfate (SDS) are efficient at solubilizing cell membranes but have been shown to disrupt important ECM proteins, resulting in changes in the mechanical properties and bioactivity of the scaffold (Jeong et al., 2021). Likewise, enzymatic treatments (e.g., nucleases) degrade leftover nucleic acids but can also damage essential ECM glycoproteins responsible for cell adhesion and signaling (Tamayo-Angorrilla et al., 2022). The other major challenge concerns the heterogeneity of the tissues that are going to be decellularized. The degree of cellular density, ECM components, and structural complexity varies between different tissues and organs. For example, softer tissues such as skin and adipose tissue are more readily decellularized than dense tissues such as cartilage or hypervascularized organs such as the liver. This variability necessitates tissue-specific protocols, posing challenges to standardization and scalability. Additionally, the vascular pattern of certain tissues is difficult to maintain during the decellularization process, while it is necessary for cell repopulation and consequent vascularization. (Moffat et al., 2022; Tenreiro et al., 2021). Another major challenge is the use of residual cytotoxic agents. Numerous decellularization protocols require extreme chemical detergents, acids, or organic solvents that, unless completely rinsed, are commonly embedded in the ECM. These residues can hinder recellularization by inducing a cytotoxic environment for seeded cells or disrupting subsequent applications. Destructive rinsing protocols are needed, but attaining residual-free minerals can be problematic without disintegrating the ECM (Fernández-Pérez and Ahearne, 2019). Decellularization processes also induce mechanical and functional changes in the ECM, which may affect scaffold performance. Alterations in stiffness, elasticity, or porosity can disrupt the natural signals required for cell proliferation and differentiation. Such changes may limit the ability of the scaffold to endure physiological loads, particularly in applications such as organ regeneration (Liao et al., 2008; Franko et al., 2024) (Figure 1). At the translational level, scaling decellularization methods for large, complex tissues and organ systems poses substantial challenges. Successful clinical translation and regulatory approval depend on consistency, repeatability, and compatibility across batches. Nonetheless, intrinsic discrepancies in donor tissue characteristics, treatment duration, and procedural parameters present considerable obstacles to uniformity. Furthermore, the high cost and labor-intensive nature of the decellularization procedure prevent it from being widely used in clinical settings. To address these constraints, the advancement of automated, high-throughput systems and refined processes is essential. These developments will improve the scalability, cost-effectiveness, and accessibility of decellularized scaffolds, hence promoting their wider use in regenerative medicine and treatments (Somasekhar and Griffiths, 2023; Hamilton et al., 2022).

## 3 Recellularization and limitations

The recellularization of decellularized tumor tissues is an essential process in the development of three-dimensional cancer models (Sensi et al., 2020). This process encounters substantial difficulties that impede its effectiveness, such as unequal cell distribution, poor cell viability, and insufficient vascularization (Ott et al., 2008; Stoian et al., 2024). A fundamental issue in recellularization is attaining homogenous cell distribution throughout the decellularized matrix. Studies have indicated that variable porosity and altered ECM composition may hinder cellular infiltration and adhesion, leading to aberrant tissue regeneration and an inability to accurately duplicate native tissue architecture. Despite advancements in seeding techniques, researchers have observed variability in cell migration, particularly in the denser regions of the scaffold (Cassani and Olson, 2018; Xu M. S. et al., 2024). Interestingly, Dong et al. reported that decellularization of a tissue can affect its porosity and lead to poor cell infiltration and adhesion. They modified the decellularized scaffold with acetic acid to increase the pore size of the ECM to achieve successful recellularization (Dong et al., 2009). One of the major challenges in the field of tissue engineering is maintaining the viability and function of cells implanted in bio-scaffolds. The other point is that the removal of key bioactive molecules, including cytokines and growth factors, can lead to a decrease in structural integrity and cell survival in the implanted environment. Recent findings have demonstrated that the lack of these critical components markedly disrupts cellular metabolism and communication inside the scaffold (Liu et al., 2022; Ebrahim et al., 2021). In a study performed by Choi et al., decellularized ECM modified with essential cytokines or chemokines such as SDF-1α improved the outcome of recellularization (Choi et al., 2025). One of the most important limitations in recellularization is the lack of functional vascular networks within the scaffold. Scientists have reported that insufficient vascularization leads to hypoxia and nutrient deficit in cells in deeper regions of the matrix, resulting in reduced proliferation and increased death (Singh et al., 2013). Alternative methods include coculturing with endothelial cells and pre-vascularization techniques to improve recellularization; nevertheless, the results indicate that these approaches are insufficient for establishing enduring functional vasculature (Fathi et al., 2021; Luque-Badillo et al., 2024). Some of the novel approaches used for recellularization are classified in Table 2.

**TABLE 2:** Recellularization methods.

Method	Protocol	Advantages	Disadvantages	Ideal for	Reference
Dynamic bioreactor systems	Perfusion of media through scaffolds under controlled conditions to mimic physiological flow	Uniform cell distribution; enhanced oxygen/nutrient delivery; promotes ECM remodeling	Complex setup; high cost; requires optimization for each tissue type	Large organs, dense tissues (e.g., heart, liver)	Lee et al. (2021)
Preconditioning with ECM coatings	Application of ECM proteins (e.g., fibronectin, laminin) or synthetic peptides to the scaffold	Enhances cell adhesion and proliferation; restores lost bioactivity	Cost of ECM components; partial coverage of scaffold surface	Thin or soft tissues (e.g., skin, intestine)	Caires-Júnior et al. (2021), Wagner et al. (2014)
Bioactive hydrogels integration	Embedding scaffolds with hydrogels loaded with cells and growth factors	Mimics natural ECM hydration; allows even cell distribution; customizable bioactivity	Risk of hydrogel detachment; mechanical mismatch with native tissue	Tumor microenvironments; soft tissue engineering	Quinteira et al. (2024)
Gene editing of cells	Editing cells (e.g., *via* CRISPR) to enhance ECM remodeling or growth factor secretion	Tailored cell behavior; enhances scaffold integration and tissue regeneration	Ethical concerns; technical complexity; off-target effects	Personalized medicine, chronic tissue defects	Zhu et al. (2022)
Exosome/EV incorporation	Loading exosomes or EVs into scaffolds or codelivering them with cells	Improves cell-ECM interactions; promotes angiogenesis and tissue regeneration	Isolation of exosomes is time-consuming; dose optimization is challenging	Tumor modeling; vascularized tissues	Rahmati et al. (2024)
Multi-cellular co-culture systems	Sequential or simultaneous seeding of different cell types (e.g., fibroblasts, immune cells)	Mimics *in vivo* microenvironment; promotes vascularization and ECM remodeling	Complex interactions may require precise timing and ratios; higher costs	Tumor microenvironments; complex organ systems (e.g., lungs)	Xu et al. (2019)
*In* *situ* bioprinting	Direct deposition of cells and bioinks onto scaffolds using 3D bioprinting	High spatial precision; customizable patterns; supports heterogeneity	Limited scalability; bioprinting resolution depends on scaffold geometry	Hierarchical tissues; organ-specific models	Garreta et al. (2017)
Microfluidic platforms	Using microfluidic devices to deliver cells in controlled gradients or patterns into scaffolds	Precise spatial control; enables creation of heterogeneous microenvironments	Requires custom fabrication; scalability issues for large tissues	Tumor models; gradient-dependent tissue engineering	Yu et al. (2024), Gil et al. (2023)
Magnetic/Electric field stimulation	Labeling cells with magnetic nanoparticles or applying electric fields to direct cell migration	Enhances cell migration; noninvasive; potential for deeper scaffold penetration	Requires special equipment; potential safety concerns (e.g., heating, toxicity)	Dense or deep tissues; vascularized organs	Yang et al. (2022), Ribeiro et al. (2019)
Smart materials integration	Using materials that release growth factors or change properties in response to stimuli (e.g., pH)	Temporal and spatial control over cell behavior; responsive to physiological conditions	Limited commercial availability; complex fabrication processes	Dynamic tissues; long-term regeneration models	Yu et al. (2023)

## 4 Macromolecules in the tumor microenvironment

### 4.1 Proteoglycans (versican, biglycan)

Proteoglycans such as versican and biglycan are major components of the ECM and are implicated in regulating cell‒matrix interactions, the ECM architecture, and tumor progression (Papadas et al., 2020). These high-molecular-weight macromolecules consist of a central core protein to which glycosaminoglycans (GAGs) are covalently attached. An abundant proteoglycan in the tumor stroma, versican, binds to HA and other components of the ECM to improve cell migration. Additionally, Versican activates FAK/MAPK signaling *via* integrins, which can increase cell proliferation and migration (Li et al., 2023; Evanko et al., 1999).

In cancers, versican is expressed at relatively high levels and is correlated with tumor invasiveness. By binding growth factors and cytokines, versican modifies the local microenvironment to promote tumor cell proliferation, migration, and immune evasion. Furthermore, during metastasis, ECM remodeling is essential, and versican plays an important role in this process by modulating ECM mechanics, facilitating the mechanical plasticity required for tumor cell motility and invasion (Mitsui et al., 2017). Another structurally important proteoglycan is biglycan, which contributes to ECM organization and plays a role in cell adhesion. The TGF-β-activated Smad pathway plays an essential role in ECM remodeling and fibrosis, leading to increased tumor stiffness and invasion, as demonstrated by the ability of biglycan to bind TGF-β, both of which have been previously implicated in tumor promotion and cancer prognosis (Kolb et al., 2001; Diehl et al., 2021).

A decellularized ECM can disrupt the ability of tumor cells to reestablish adhesion and migrate since the degradation of proteoglycans cannot fully maintain ECM integrity during the decellularization process. Thus, replacing versican, biglycan, and other proteoglycans into a decellularized scaffold might promote the recellularization process, allowing tumor cell migration and functionality in the tumor model (Mendibil et al., 2020; Kinsella et al., 2004).

### 4.2 Collagen

Collagen (28 identified types in human) is the most abundant protein in the ECM and serves as a critical structural component, providing tissues with mechanical stability and support. Within the TME, collagen fibers form a complex scaffold that not only sustains tumor cells and stromal components but also actively participates in tumor progression (Salvatore et al., 2021). Among the various types of ECM collagen, type I and III collagens are predominantly expressed by cancer-associated fibroblasts (CAFs), which are key players in shaping the tumor stroma. These collagens contribute to stromal stiffness, an important factor in modulating cancer cell behaviors such as invasion, metastasis, and resistance to therapy (Wright et al., 2023; Wang Z. et al., 2024). Integrins on the tumor cell surface bind collagen to activate pathways promoting cell survival, migration, and proliferation. A primary pathway that is activated following collagen–integrin binding is the focal adhesion kinase (FAK)/Src signaling pathway. FAK autophosphorylation excites Src family kinases that phosphorylate adaptors such as paxillin and talin. The cytoskeleton is rearranged as a result of these events, enabling the migration and invasion of cells (Van Slambrouck et al., 2007; Fatherree et al., 2022; Mammoto et al., 2013). Additionally, the FAK/Src pathway activates downstream mediators, such as Rho GTPases (RhoA, Rac1, and Cdc42), that control actin polymerization and lamellipodia formation, which are essential for motility (Hernández et al., 2018). Collagen–integrin binding concurrently activates the PI3K/AKT pathway. Integrin clustering recruits and activates PI3K (phosphatidylinositol 3-kinase), generating PIP3, which subsequently activates AKT. The AKT pathway phosphorylates key substrates involved in cell survival (e.g., GSK3β), growth (e.g., mTOR) and migration (e.g., BAD), suppressing apoptosis and promoting metabolic activity. This signaling cascade suppresses apoptosis and promotes metabolic activity, facilitating the proliferation and migration of cancer cells under adverse conditions (Pungsrinont et al., 2021; Wu et al., 2019). Interactions between collagen and integrins activate the MAPK/ERK pathway (mitogen-activated protein kinase). FAK and Src activation activate the Ras-Raf-MEK-ERK pathway, leading to ERK1/2 phosphorylation. Upon activation, ERK translocates to the nucleus, where it modulates transcription factors such as AP-1, resulting in the expression of genes that promote proliferation, migration, and angiogenesis (Peng and Gassama-Diagne, 2017; Dedieu and Lefebvre, 2006; Chetoui et al., 2006). Additionally, collagen has been shown to promote YAP/TAZ activation, in part *via* mechanical engagement and matrix stiffness. Integrins transmit extracellular physical signals to the cytoskeleton in a manner that induces YAP/TAZ nuclear translocation, where they act as transcriptional coactivators to increase the expression of genes that promote tumor progression (Sapudom et al., 2025). Moreover, collagen‒integrin interactions modulate NF-κB activity, promoting inflammation, cell survival, and metastasis. NF-κB activation upregulates cytokines, MMPs, and antiapoptotic proteins, further driving cancer progression. Type I collagen, often overexpressed in tumors, stiffens the ECM, promoting tumor progression and resistance to therapy. Despite its structural role in the ECM, collagen is partly removed or changed during decellularization and typically remains in variable amounts especially type I and III. However, decellularization alters its 3D structure and molecular organization, affecting its interactions with cells. These structural changes influence the behavior of tumor cells that recellularize into the scaffold, altering their proliferation and migration (Liu et al., 2024; Mancini et al., 2024). Therefore, the reintroduction of collagen into decellularized matrices is required for enhancing cell attachment, restoring functional ECM, and promoting a native-like TME structure (Martinello et al., 2014; Ghosh et al., 2022).

### 4.3 Fibronectin

Fibronectin is another important ECM glycoprotein that plays a vital role in cell adhesion, migration, and ECM remodeling. During tumor stroma development, fibronectin is highly expressed in the stroma, where it maintains the structural integrity of the ECM. As a bridge between integrins and other ECM components, fibronectin promotes tumor cell adhesion and motility. In the tumor environment, fibronectin facilitates cancer cell migration during metastasis and increases tumor stroma stiffness, a tunable property with significant relevance in cancer progression (Han and Lu, 2017). The interaction between α5β1 and αvβ3 integrins on tumor cells and fibronectin initiates FAK/Src signaling pathways that induce cytoskeletal remodeling and focal adhesion formation, thereby promoting cancer cell metastasis (Li et al., 2015). Notably, fibronectin polymerization with collagen contributes to ECM stiffening *via* cross-linking mechanisms mediated by lysyl oxidase (LOX) (Zhao et al., 2009). This increased stiffness activates mechanotransduction pathways through YAP/TAZ signaling, which can further drive cancer development. The dual functions of fibronectin in ECM turnover and enhanced metastasis are therefore critical in the context of tumor progression (Raghunathan et al., 2013). Some evidences indicated that decellularization process with alkaline-pH often leads to the removal of fibronectin from the tissue, resulting in insufficient support for cell attachment within the decellularized scaffold. This loss limits the migration of tumor cells and their proliferation during recellularization, which is why fibronectin is reintroduced into the scaffold. Fibronectin is a cellular adhesion factor and can help improve aspects of cells that are important during recellularization and establish a functioning tumor model (Fu et al., 2014; Afrin et al., 2025; Tsuchiya et al., 2014). Moreover, fibronectin interacts with other ECM components, such as collagen and HA, and reinforces the framework and role of the TME (Di Vito et al., 2024).

### 4.4 Hyaluronic acid

HA is a polysaccharide that is another component of the ECM in various tissues, including tumors. HA plays multiple roles in the TME, such as cell migration, ECM remodeling, and tissue repair (Chanmee et al., 2016). Increased HA levels in tumors are frequently associated with cancer progression, as HA-rich matrices increase ECM deposition, creating a microenvironment conducive to cancer cell invasion. Furthermore, HA modulates the mechanical characteristics of the ECM, such as tissue stiffness, which directly impacts tumor cell behavior (Gagneja et al., 2024; Suo et al., 2019). During decellularization, HA is often eliminated, resulting in the loss of these important functions, such as the regulation of tumor cell adhesion and migration. It is also possible that HA is depleted, leading to less flexible and less permissive ECM for recellularization (Uhl et al., 2020). The addition of HA to decellularized tissues can also enhance matrix mechanical properties and aid in cellular retention and migration, thus improving recellularization (Su et al., 2014). In addition, HA binds to cancer cell surface receptors such as CD44 and RHAMM and activates downstream signaling pathways, including the PI3K/AKT and MAPK pathways, which regulate the processes of proliferation, survival, migration, and invasion (Misra et al., 2015). Recent studies have shown that RHAMM interacts with HA and ECM proteins such as fibronectin to play a role in tumor cell migration as well as modulating tumorigenesis (Michalczyk et al., 2022). HA modifies stroma and immune cells, leading to immune evasion within the TME and promoting tumor progression. The high-molecular-weight form of HA preserves ECM integrity, whereas low-molecular-weight fragments elicit a proinflammatory response and angiogenesis. Furthermore, HA-rich ECM elevates interstitial pressure, obstructing drug access and promoting therapeutic resistance (Price et al., 2018).

## 5 Proteins essential for tumor cell behavior and recellularization

### 5.1 Laminin and newly discovered variants

Laminin is the key structural component of the basement membrane, which is well known for its involvement in the TME, specifically in the modulation of cell activity (Nonnast et al., 2024). The novel functional roles of laminin isoforms during cancer progression have recently been recognized. For example, laminin-411 (α4β1γ1) is upregulated in aggressive cancers and is correlated with increased migration and metastasis of tumor cells (Iwamuro et al., 2021). Integrin signaling mediated by this variant allows for the adaptation of cancer cells to more advanced stages of the disease while favoring the survival and migration of tumor cells (Ishikawa et al., 2014). In addition, recent reports indicate that the interaction of laminin-511 (α5β1γ1) with CD44 is involved in epithelial‒mesenchymal transition (EMT) in several types of cancer cells. These findings suggest that targeting this interaction could improve ECM remodeling and the recellularization of decellularized scaffolds. Laminin-411 and laminin-511, among other isoforms, can be reintroduced into decellularized matrices to promote tumor cell adhesion and migration (Iwamuro et al., 2021; Qin et al., 2017; Caires-Júnior et al., 2021). These laminin variants have potential for ECM restoration, facilitating the recellularization of decellularized tumor scaffolds to better mimic the native TME. In addition, in tumor models, laminin provides a supportive ECM for cancer cell–stromal cell interactions while preserving both the architectural integrity and biochemical cues of the TME. The bioactivity of 3D bioprinted scaffolds has been enhanced *via* the use of laminin-based bioinks, which promote tumor-specific ECM deposition and cell viability. These scaffolds have great potential for simulating tumor behavior *in vitro*, which is relevant for drug screening, mechanistic studies, and precision medicine research (Mi et al., 2023; Toshmatova et al., 2019; Ma et al., 2020).

### 5.2 Fibronectin-related protein, tenascin-C

Although fibronectin has previously been investigated with respect to the TME, findings have shown that other ECM components, such as tenascin-C, are also important for ECM remodeling and cancer progression (Hartmann et al., 2025). TNC is a glycoprotein that is upregulated in tumors, particularly within the stromal compartment, and promotes tumorigenesis and metastasis indirectly by increasing interactions with integrins and other ECM components (Yoshida et al., 2015). Recent studies have demonstrated that it can modulate both the rigidity and elasticity of the ECM while regulating cancer cell behavior in 2D and 3D models (Kang et al., 2016). It enhances cancer cell migration and contributes to tumor-associated inflammation, making it a key factor in recreating an effective TME (Orend and Chiquet-Ehrismann, 2006). Mechanistically, TNC promotes tumor progression through integrin- and Toll-like receptor (TLR)-mediated signaling pathways (Marzeda and Midwood, 2018). TNC interacts with integrins (α9β1 and αvβ3) on cancer and stromal cells, activating the FAK, PI3K/AKT and MAPK pathways for cell proliferation and migration (Shi et al., 2015; Zhang et al., 2021; Zhu et al., 2020). Notably, TNC interacts with TLR4, leading to NF-κB activation, which increases the inflammatory response and immune evasion (Zuliani-Alvarez et al., 2017). TNC also modulates EMT and angiogenesis through growth factor regulation (e.g., VEGF). Its presence in the TME promotes tumor progression, immune suppression, and therapy resistance (Katoh et al., 2013; Sun et al., 2025). Overall, in terms of the role of TNC, its recolonization of decellularized tumor tissues can potentially recreate an active ECM, leading to improved recellularization and potentially a more accurate model of a tumor (Gilpin et al., 2017; Bhattacharya et al., 2024).

### 5.3 Periostin

Periostin, an ECM protein known to interact with integrins and other ECM components, is also newly recognized as a facilitating agent in tumor progression and metastatic spread. Its involvement in ECM remodeling and its pivotal role in tumor angiogenesis, invasion, and chemotherapy resistance have been extensively documented (Dorafshan et al., 2022). Periostin also interacts with different tumor-associated cells, such as CAFs and immune cells, to assist in the remodeling of the tumoral environment. Periostin induces the integrin/ERK/NF-κB axis in cancer cells through an autocrine mechanism that causes the secretion of different cytokines, including MIP-1β (macrophage inflammatory protein-1 beta), MCP-1 (monocyte chemoattractant protein-1), TNFα (tumor necrosis factor-alpha), and RANTES. These cytokines promote the recruitment and chemotaxis of THP-1 monocytes, which can then polarize to M2 macrophages *in vitro*. Similarly, compared with control tumors, tumors derived from Periostin-overexpressing SKOV3 cells (a human ovarian cancer cell line) exhibited significantly greater infiltration of tumor-associated macrophages (TAMs). On the other hand, Periostin promoted TGF-β2 secretion by ovarian cancer cells, activating adipose-derived stromal cells, which were transformed into CAF-like cells expressing alpha-smooth muscle actin (αSMA) and fibroblast activation protein alpha (FAP-alpha). This correlates with the increased presence of CAFs in metastatic tumors originating from periostin-expressing SKOV3 cells (Lin et al., 2022; Fan et al., 2025). It has been proven that periostin binds to integrins on CAFs (e.g., αvβ3, αvβ5, and α6β4) to activate the FAK/PI3K/AKT and MAPK signaling pathways, which mediate CAF activation, proliferation, and the secretion of ECM components such as collagen and fibronectin. These factors promote the strengthening of the tumor stroma and stiffer tissue, which also promotes the migration and invasion of tumor cells (De Oliveira Macena et al., 2025). In addition, periostin regulates immune cells in the TME. It also inhibits T-cell infiltration and function, contributing to an immunosuppressive environment. Periostin expression is upregulated in various cancers and is correlated with poor prognosis. More recently, periostin has been identified as a linker between ECM components, enhancing cell signaling and promoting tumor cell survival and migration (Chen et al., 2024). The incorporation of periostin into decellularized tumor matrices may restore ECM cellularity and mechanical properties, aiding in greater recellularization. The incorporation of periostin into decellularized scaffolds may also be beneficial for reestablishing interactions between CAFs, endothelial cells, and tumor cells, thereby enhancing the tumor-like characteristics of the recellularized tissue (Michaylira et al., 2010; Gillot et al., 2022).

### 5.4 Integrins and ligands

Integrins are transmembrane receptors that facilitate the general and specific adhesive functions of cells to components of the ECM. These receptors play key roles in the control of cell migration, survival, and differentiation (Chastney et al., 2024). These receptors facilitate both the general and specific adhesive functions of cells toward ECM components. Integrins are heterodimers of α and β subunits, each with binding specificities for different ligands, such as αvβ3 and α5β1 to fibronectin, α6β4 to laminin, and α2β1 to collagen. Integrin dysregulation in cancer drives tumor cell invasion and metastasis, commonly through crosstalk with growth factor receptors. Integrin signaling is mediated mainly by the FAK and Src pathways, which activate downstream effectors such as PI3K/AKT, Rho GTPases, and MAPKs. These cascades promote cytoskeletal remodeling, proliferation, and EMT. Moreover, integrins induce angiogenic signaling *via* VEGF activation and the regulation of stiffness in the TME (Cooper and Giancotti, 2019).

### 5.5 Matrix metalloproteinases

MMPs constitute a group of enzymes responsible for the degradation and remodeling of ECM components. These processes are essential not only for ECM homeostasis but also for cancer cell migration and angiogenesis. MMPs, such as MMP-2, MMP-9, and MMP-14, are upregulated in tumors and promote tumor invasiveness *via* degradation of the ECM, facilitating the migration and invasion of carcinoma cells into surrounding tissues (Westin and Rabelo-Santos, 2015; Wang et al., 2025; Xu W. X. et al., 2024). Studies have shown that MMPs promote the remodeling of decellularized ECM to resemble the native TME more closely. By reintroducing MMPs into the decellularized scaffold, the recellularization process can be further improved by permitting cancer cells to remodel the ECM as they re-establish themselves in the tissue (Sehic et al., 2024).

### 5.6 Growth factors (e.g., VEGF, FGF, and TGF-β)

Growth factors are signaling molecules that modulate cell proliferation, differentiation, and survival. Tumor growth factors within the TME, such as vascular epithelial growth factor (VEGF), fibroblast growth factor (FGF), and transforming growth factor-β (TGF-β), are some of the factors that drive tumor growth and metastasis. VEGF activates angiogenesis to develop a vasculature network, ensuring nutrient supply to the growing tumor. FGF stimulates tumor cell proliferation and angiogenesis. TGF-β is involved in EMT, which enhances cell motility and invasiveness (Jiang et al., 2020; Yan and Shi, 2024; Turner and Grose, 2010). During recellularization, these growth factors may no longer be available to the tumor cells, as they may have been eliminated and/or degraded during the decellularization process and not retrieved. To induce the TME in recellularized scaffolds, growth factors must be reintroduced to promote tumor cell proliferation, migration, and angiogenesis. This allows the decellularized matrix to better mimic the natural tumor environment, thereby improving the accuracy of the cancer model and facilitating its use in drug screening and other experiments (Wu et al., 2022; Conklin et al., 2004).

### 5.7 Secreted protein acidic and rich in cysteine (SPARC)

SPARC, or osteonectin, is a matricellular protein that binds to components of the ECM (Ghanemi et al., 2020a). In addition to regulating ECM remodeling, SPARC regulates cell adhesion, migration, and differentiation. Within the TME, SPARC is involved in modulation of the ECM, promoting tumor cell growth and invasion. On the other hand, SPARC reportedly interacts with integrins and MMPs, remodeling the ECM and increasing cell motility. SPARC is frequently overexpressed in tumors and is associated with poor prognosis and increased aggressiveness (Ghanemi et al., 2020b). In doing so, by loading SPARC into decellularized tumor tissues, the recellularization process could be enhanced to allow cells to remodel the ECM in a more effective manner (Kerr et al., 2024).

## 6 Strategies for improving recellularization

High-throughput approaches, in relation to the subject matter of this review, are meant to describe experimental systems that facilitate, in parallel, the creation, recellularization, and functional assessment of many variants of tumor environments within an efficient, scalable, and reproducible fashion. As opposed to efforts aimed at optimizing scaffolds under one set of conditions, high-throughput strategies outline methods in which the ECM composition, cellular components, and biochemical signals are varied under several conditions. Microfluidic array systems, perfusion bioreactors, as well as 3D bioprinting-based combinatorial systems are high-advanced strategies covered in the following paragraphs.

### 6.1 Importance of ECM‒cell interactions for effective recellularization

Successful recellularization of decellularized tumor tissue requires preservation or restoration of crucial ECM–cell interactions, which regulate a variety of cell functions, including adhesion, migration, differentiation, and survival. As discussed, ECM components, along with newly identified molecules such as periostin and TNC, are prevalent in the TME and significantly influence tumor cell behavior. Embedding the molecules into decellularized scaffolds significantly improved the adhesion and migration of cells and, consequently, their recellularization (Sohn et al., 2020; Bhattacharya et al., 2024). This stage includes the inoculation of tumor cells, fibroblasts, and other relevant cell types into the decellularized scaffold. These proteins interact with the ECM, and their proliferation and organization in the scaffold depend on the integrity of the ECM proteins and their interaction with cell surface receptors (such as integrins and CD44) (Hillebrandt et al., 2019; Weidenhamer et al., 2015). By reintroducing critical ECM components such as laminin-111, fibronectin, and HA, a more supportive environment is offered for tumor cells to recreate their matched/native microenvironment. One method to achieve this restoration is through the coating or embedding of these proteins in scaffolds or bioinks used for 3D bioprinting (Toshmatova et al., 2019; Badylak et al., 2009; Chen et al., 2022).

Recent studies suggest the use of biomaterials that closely resemble the mechanical properties of the tumor ECM, including hydrogels and decellularized tissue-derived materials, for enhanced recellularization. These materials allow for cell growth, preserve the tumor-like characteristics of the scaffold, and cause the tumor cells to behave in a similar fashion to what they would do in the native TME (Barthold et al., 2021). Moreover, the application of signaling molecules, growth factors, or cytokines can drive cell behavior to increase recellularization efficiency (Wen et al., 2016). Therefore, the combination of ECM structural proteins with other essential factors represents an attractive strategy for recapitulating tumor conditions and facilitating successful recellularization (Badylak et al., 2011).

### 6.2 3D bioprinting

**FIGURE 2:**
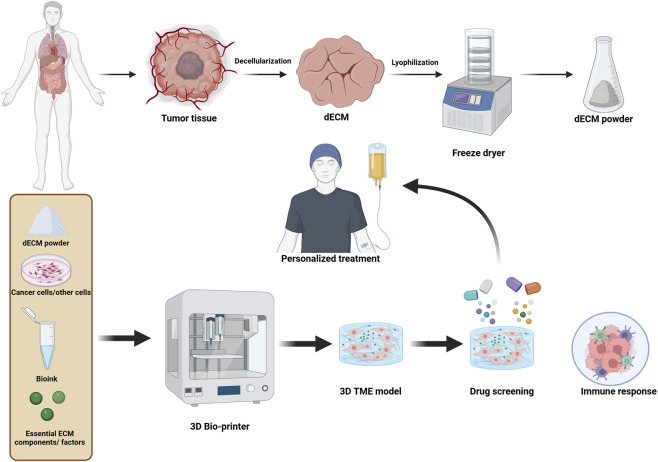
Schematic illustration of the design of personalized cancer therapy with a 3D bioprinter: patient-derived tumor tissue is decellularized and lyophilized to obtain dECM powder. It is then homogenized with bioink, cancer cells, ECM components, and other factors to create a 3D TME *via* a 3D printer. This model allows for drug screening and immune response assessment and forms the basis for personalized therapeutic approaches.

Currently, 3D bioprinting provides a versatile platform for fabricating complex tissue models that reflect the TME. This high-throughput technology facilitates the accurate placement of various cell types and ECM proteins within the scaffold to develop functionally relevant and organized tissues (Zhao et al., 2014; Guo et al., 2024). Recent advancements in bioprinting technologies have enabled the construction of tumor models that more closely mimic native tumors in both structure and functionality. Therefore, the 3D bioprinting technique enables the formation of tumor tissues with high fidelity by integrating decellularized ECM scaffolds and tumor-derived cells. The model fidelity of the tumor can be enhanced by the addition of macromolecules and specific proteins, such as laminins, collagens, and fibronectin, to the bioink formulations. Furthermore, printing multiple cell types, such as cancer cells, cancer-associated fibroblasts (CAFs), endothelial cells, and immune cells, can create a more comprehensive TME (Gopinathan and Noh, 2018; Grottkau et al., 2024; Sup Yoon et al., 2024). With respect to bioinks, hydrogels based on decellularized tumor tissue or ECM proteins (e.g., fibrinogen and HA) can significantly improve cell viability and recellularization. These hydrogels not only support the structural integrity of tumor cells but also encourage spatiotemporal migration and proliferation of tumor cells (Gungor-Ozkerim et al., 2018). These technologies include multilayer and laser-assisted printing, which enable the production of complex and heterogeneous 3D tumor models. The coorganized ECM deposition with cell bioprinting provides powerful advantages in constructing a more physiologically relevant TME that recreates tumor behaviors and drug responses *in vivo* in order to personalize treatment (Figure 2) (Yang et al., 2021; Shukla et al., 2022).

### 6.3 Growth factors and cytokines for enhanced recellularization

The addition of these growth factors (VEGF, TGF-β) will aid in guiding the migration, proliferation, and organization of tumor cells and fibroblasts within the scaffold in the case of the recellularization of decellularized tumor tissue (Li et al., 2024). Furthermore, immune-modulatory cytokines such as interleukins (such as IL-6 and IL-8) can modulate the immune response in the TME. These cytokines drive the recruitment and activation or suppression of immune cell types (with dual functions in the TME), potentially restoring the immune microenvironment in recellularized tumor tissues (Habanjar et al., 2023). From the viewpoint of the immune response, including these cytokines and growth factors in decellularized scaffolds ultimately promotes the functionalization of recellularized tissues and their ability to mimic the native TME (De Rubis et al., 2024).

Controlled-release systems for the delivery of growth factors and cytokines within recellularized tissues have also been explored in recent studies. For example, these materials may be incorporated in nanoparticles or microparticles to be released over time to ensure that the material remains effective at influencing concentrations within the scaffold. This approach not only improves cell behavior but also promotes a more active TME (Fathi-Achachelouei et al., 2019).

### 6.4 Microfluidic systems

Microfluidic systems are a kind of cutting-edge strategy that can be used for recellularization, allowing for the manipulation of cell seeding and nutrient delivery, as well as tissue microenvironments with high spatiotemporal resolution (Ghanbari et al., 2021). This microlab on a chip is engineered *via* microscale channels, often made from biocompatible materials such as polydimethylsiloxane (PDMS), enabling the precise manipulation of fluid flow. Load target cells, such as fibroblasts, epithelial cells, or immune cells suspended in a medium, into the microfluidic device. Within the decellularized scaffold, uniform cell seeding is achieved by controlling the flow rates and pressure gradients (Mahhengam et al., 2022; Hashemzadeh et al., 2020). Importantly, owing to the laminar flow behavior unique to microfluidics, there is low turbulence, allowing the cells not only to attach simply but also to penetrate deep into the pores of the scaffold. Once the cells are seeded, microfluidic devices allow dynamic perfusion of culture medium, oxygen, and growth factors to keep the cells alive and encourage their proliferation. These systems are capable of replicating physiological conditions such as mechanical shear stress and biochemical gradients that improve cell functionality, differentiation, and scaffold integration (Tong and Voronov, 2022). In addition, microfluidic platforms can be used to coculture multiple cell types to recreate a sophisticated tissue microenvironment, especially in the cancer field. The integrated sensors and imaging tools provide real-time monitoring of recellularization efficiency, cell distribution, and viability, guaranteeing accurate assessments of recellularization efficiency (Xu H. et al., 2024).

Microfluidic systems have significant advantages. These techniques allow for fine control of the dynamic recellularization process, enhancing both cell distribution and scaffold colonization relative to static seeding approaches. The systems recapitulate in vivo-like mechanical and biochemical stimuli, with the latter promoting tissue-like properties and cell differentiation. Moreover, their dynamic perfusion characteristics stimulate cell proliferation and integration, and high-throughput designs facilitate the simultaneous processing of several scaffolds for reproducibility and scalability (Toley et al., 2011; Ko et al., 2024).

However, microfluidic systems have their own limitations. Their design and operation are highly technical, and this poses a barrier to widespread adoption. The cost associated with microfabrication tools and components further complicates this issue, especially in resource-limited environments. Another common concern is scalability because the transfer of microfluidic protocols to large tissue constructs is technically demanding. Second, during dynamic seeding, high shear stress may also decrease cell functionality if the shear is not properly controlled. The absorption characteristics of PDMS could also impair the delivery of certain biomolecules due to material constraints (Halldorsson et al., 2015).

**FIGURE 3:**
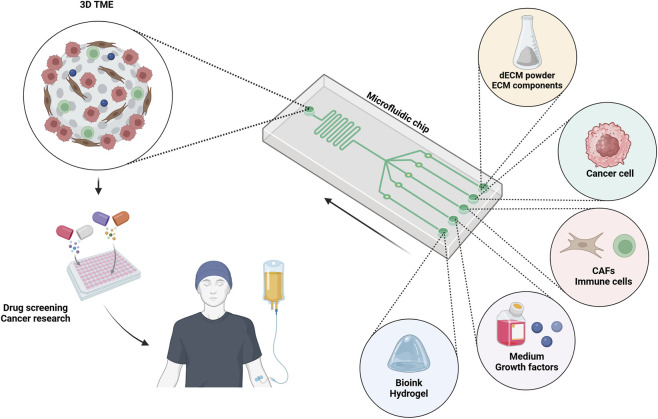
This schematic image shows how a microfluidic platform can improve the process of recellularization as well as the creation of a 3D tumor microenvironment for clinical research.

Overall, the flexibility of this technology with respect to the incorporation of biomarkers has made microfluidic systems ideal candidates for recellularization methods. They are propelling advances in tissue engineering, TME modeling, and regenerative medicine by enabling spatially controlled, dynamic perfusion, and microcellular coculture capabilities. Overcoming existing barriers will unlock their potential for clinical and research applications (Figure 3) (Xie et al., 2021).

### 6.5 Bioreactors

Bioreactors are another advanced device developed to maintain a dynamic and regulated atmosphere for the recellularization of decellularized scaffolds (Bonvillain et al., 2013). Unlike static culture methods, bioreactors integrate mechanical, biochemical, and hydrodynamic stimuli that promote the even distribution of cells, increase cell viability, and promote tissue maturation. These systems are essential for tissue engineering, as they emulate physiological conditions and address diffusion limitations frequently faced in static cultures (Bilodeau and Mantovani, 2006). The basic function of bioreactors includes coupling fluid flow to perfuse cell-laden media through or around the decellularized scaffold. A precisely regulated flow of fluids guarantees a sufficient supply of oxygen and nutrients, as well as efficient removal of metabolite waste products favorable for cell growth (Schmid et al., 2018; Ahmed et al., 2019). Today, various types of bioreactors are operational in laboratories, some of which include perfusion bioreactors, spinner flasks, and mechanical stimulation bioreactors. Perfusion bioreactors show particular promise for recellularization because they cause uniaxial transport of the cell suspension into the scaffold pores under controlled pressure gradients, promoting deeper cell penetration and homogenously distributed cell ingrowth (Hansmann et al., 2013). Mechanical stimuli, such as shear stress, compression, or tensile strain, activate mechanotransduction pathways, influencing cell behavior and tissue-specific matrix deposition (Dermenoudis and Missirlis, 2010). For example, cyclic strain in cardiovascular bioreactors promotes vascular smooth muscle cell alignment, whereas shear stress in cartilage models promotes chondrocyte functionality. This specific mechanobiological conditioning accelerates tissue development, producing constructs that closely resemble native tissues (Schutte et al., 2010; Schulz and Bader, 2007). Bioreactors offer significant advantages, including overcoming diffusion barriers, promoting homogeneous cell proliferation in the scaffold, and allowing real-time monitoring of essential variables (temperature, pH, oxygen tension, and flow rates). They also enable the coculture of multiple cell types, supporting the creation of complex engineered tissues, such as models of the TME. However, bioreactors face several challenges, including the need for knowledge, high costs, and difficulties in scaling up to larger tissue constructs. Achieving uniform cell distribution and adequate mechanical conditioning at large scaffold sizes remains challenging, and uncontrolled flow rates or mechanical stresses may damage cells, reducing their recellularization efficiency (Mandenius, 2016; Niu et al., 2023; Mousavi Shaegh et al., 2016).

## 7 Controversies and standardization challenges

Despite the increased interest in decellularized tumor models, there exist some remaining controversies that still affect the standardization of methods as well as comparison between studies. These include detergent-based decellularization methods, such as the comparison of the efficiency of SDS and Triton X-100. Although it has been shown consistently that SDS exhibits an efficiency in cellular and DNA removal, significant loss of crucial ECM components, such as glycosaminoglycan, laminin, and fibronectin, has been identified in multiple studies. However, these methods preserve ECM ultrastructure and bioactive ECM proteins more efficiently than SDS, although with reduced decellularization efficiency, especially within cellular or dense tumor tissue (Lü et al., 2014; Hoshiba, 2019; White et al., 2017). Notwithstanding, an important point is that the differences in ECM retention have been observed to be affected by various concentrations, times, tissue thickness, as well as wash methods in multiple studies (Crapo et al., 2011).

Variability in decellularization method also poses a challenge that is exacerbated by differences in outcomes based on the type of tumor for recellularization. For example, decellularized matrices derived from solid tumors such as colorectal or breast carcinomas exhibit efficient cell adhesion and infiltration, whereas tumors that are either too rigid or are of a complex architecture such as pancreatic or brain tumors may present restricted heterogeneity in recellularization. Such differences emphasize that recellularization is highly dependent on ECM properties in a tissue-specific manner (Marques-Magalhães et al., 2022; Henke et al., 2020).

Collectively, these inconsistencies draw attention to the lack of standardized criteria for evaluating decellularization efficacy, ECM preservation, and recellularization success. The utilization of variable criteria in individual studies has resulted in inconsistent outcomes within the literature. These issues and challenges can only be addressed by implementing standardized criteria for evaluating success and optimizing for tumors.

### 7.1 Tumor-origin–dependent technical variability in decellularized models

Outcomes of decellularization and recellularization are highly dependent upon the type of tumor due to intrinsic variations in architecture, ECM composition, and cellularity. Decellularized tumor systems are generally more relevant to solid tumors due to the presence of a defined ECM scaffolding which can support complementary biochemistry (Hoshiba, 2019; Iazzolino et al., 2022). In contrast, hematologic malignancies do not possess an ECM-based cohesive tissue architecture, making such tumors poorly suited for decellularization strategies and, by extension, require different methodologies encompassed in non-scaffold formats (Huang et al., 2021).

Even among solid tumors, there is considerable technical variation depending on tissue of origin and histological type. Cancers of epithelial origin, such as breast, colorectal, and liver cancer, are expected to have well-organized basement membrane constituents, high levels of collagen networks, and well-demarcated stromal regions. Such features are known to favor effective decellularization as well as efficient recellularization of cancer cells and stromal cells. For instance, decellularized matrices of colorectal and breast cancer have been shown to support effective cancer cell adhesion and maintenance of cancer phenotypes (Marques-Magalhães et al., 2022; Jin et al., 2019; Van Tienderen et al., 2023).

However, tumors that exhibit strong mesenchymal markers or extreme stromal remodeling are more challenging. This is because liver tumors tend to have high vascular complexity and region-specific ECM heterogeneity. Thus, perfusion decellularization is needed. Additionally, tumors that have high fibrosis or stiffness may pose an impedance to cell infiltration during recellularization. Such tumors may also have repopulation heterogeneity. The above points highlight that decellularization efficacy, preservation of ECM, and recellularization efficacy also vary and have to be optimized based on tissue type (Van Tienderen et al., 2023; Roy et al., 2023; Ning et al., 2023).

### 7.2 Tumor-specific recellularization in primary and metastatic ECM

Efficiency of recellularization and cellular behaviors in a decellularized tumor matrix are often dependent on the type and stage of underlying tumors. Specifically, in breast cancer, the ECM derived from primary tumors can support efficient adhesion and migration of cancer cells, whereas ECM derived from metastatic tumors can have increased cross-linking of collagen and different compositions of the basement membrane (Machado Brandão-Costa et al., 2020; Wishart et al., 2020). In colorectal cancer, the ECM within primary tumors retains spatial biochemical patterns that support epithelial tumor cell organization, whereas the ECM of metastatic sites, especially liver metastases, may show increased stiffness and remodeling patterns affecting recellularization and immune cell distribution (Marques-Magalhães et al., 2022). Renal cancer models also demonstrate the characteristics of tumor-specific limitations, with a decellularized kidney ECM exhibiting a high degree of vascular specialization and regional heterogeneity of the ECM. Such properties would differentially impact the recellularization processes in the primary *versus* the metastatic settings. Thus, a tumor-specific strategy for recellularization would be required (Bond et al., 2021).

## 8 Conclusion

Despite the success of decellularization and recellularization, there are still hurdles that must be overcome for these techniques to successfully create models of tumors. One of the key issues is preserving the integrity of the ECM throughout the decellularization process, which is one of the most crucial issues in organ decellularization. Despite improvements in decellularization protocols over time, maintaining the native structure or function of ECM components without damaging them is still a considerable hurdle. Moreover, engineered scaffolds must support effective recellularization while their cellular constituents are removed, maintaining the complexity of the ECM, which needs a further balanced approach.

A key challenge is that the TME is variable. Tumors are very heterogeneous, and this heterogeneity needs to be captured when you are making your decellularized scaffolds. Single-cell RNA sequencing and other novel technologies can be used to find tumor-specific markers and ECMs that are essential for developing more accurate tumor models for drug discovery (Zhu et al., 2023). Future studies need to incorporate such insights into decellularization and recellularization protocols to improve the fidelity of the TME. Tumor tissues that have been decellularized and then recellularized have substantial potential to accelerate cancer biology, drug screening, and personalized medicine. Restoration of the ECM components, growth factors, and signaling pathways that play critical roles in tumor progression is needed to develop a more accurate *in vitro* model of the TME.

In this context, while substantial advances have been made, considerable work remains toward further optimizing decellularization and recellularization strategies. Identification of the most potent ECM proteins, growth factors, and cell types in developing tumor models will require further studies. As advanced biomaterial-based platforms, including 3D bioprinting and organ-on-a-chip technologies, have been developed, they will undoubtedly play a pivotal role in addressing and overcoming these obstacles, leading to the establishment of even more sophisticated tumor models. Thus, decellularized tumor tissues serve as unique *in vitro* and *in vivo* models that help us not only understand aspects of the TME, such as heterotypic cells and their interactions, with the goal of eventually translating this knowledge into effective cancer therapies but also evaluate the efficacy of novel therapeutic agents in the expected TME.
